# East Tennessee, Virginia and Georgia Railroad Co.

**Published:** 1887-06

**Authors:** 


### EAST TENNESSEE, VIRGINIA AND GEORGIA RAILWAY CO.

*To Delegates to the American Medical Association:*

L. J. Ellis, A. G. P. A , Atlanta, Georgia, May 14th, 1887, writes: For the accommodation of delegates to the Medical Convention which convenes at Chicago, June 7th, we shall put on a special sleeper to run through to Chicago from Atlanta.

This sleeper will leave Atlanta, at 1 p. m., June 7th, arrive Chicago 6.50 p.m., June 8th.

I am authorized to say that a number of delegates from this city and the State will take up space in this sleeper, and we shall be glad to have you join the party. Also notice the following:

J. H. Griffin, of Atlanta, the Traveling Passenger Agent of Evansville Line, writes: This is to inform you that reduced rates to the above Convention will be made on the certificate plan, that is, that you must purchase your ticket to Chicago, paying therefor the regular one-way rate, and get a certificate from the agent selling you the same, stating the point from which sold, the route of the ticket, and the amount paid for it. When you arrive in Chicago, the Secretary of the Convention will indorse on your certificate that you have been in attendance on the Convention, and that will be authority for this Company to sell you a return ticket at *one-third* the regular rate.

